# Supplementary material for: A multi-scale CNN with atrous spatial pyramid pooling for enhanced chest-based disease detection
Source: PeerJ Comput Sci. 2025 Feb 17;11:e2686. doi: 10.7717/peerj-cs.2686 (PMC11888937; doi:10.7717/peerj-cs.2686)
Supplement: Supplemental Information 2 [file peerj-cs-11-2686-s002.docx]

**The specifications of our machine are as follows**

Google Colab Free version was used which has following hardware

**Hardware**

- **CPU**: Intel Xeon CPU with 2 cores.
- **GPU** NVIDIA K80,
- **RAM**: 12.72 GB of RAM.
- **Disk Space**: 50 GB of disk space is available for use.

**Software**

- **Python**: Colab runs Python 3.x.
- **Libraries**: Pre-installed libraries include TensorFlow, Keras, PyTorch, OpenCV, NumPy, Pandas, Matplotlib, and many other common machine learning and data analysis libraries.

**Our personal laptop had the following specifications**

Processor : 12th Gen Intel(R) Core(TM) i7-1260P, 2100 Mhz, 12 Core(s), 16 Logical Processor(s), base frequency 2.1 GHz and can be overclocked to 4.7 GHz

Ram : 32 GB DDr4 Ram

Storage : 512 GB NVME PCL4 SSD

Graphic Card : Intel Iris XE graphic card
