# Supplementary material for: A multi-scale CNN with atrous spatial pyramid pooling for enhanced chest-based disease detection
Source: PeerJ Comput Sci. 2025 Feb 17;11:e2686. doi: 10.7717/peerj-cs.2686 (PMC11888937; doi:10.7717/peerj-cs.2686)
Supplement: Supplemental Information 3 [file peerj-cs-11-2686-s003.docx]

**Description of models used:**

Our architecture is as follows


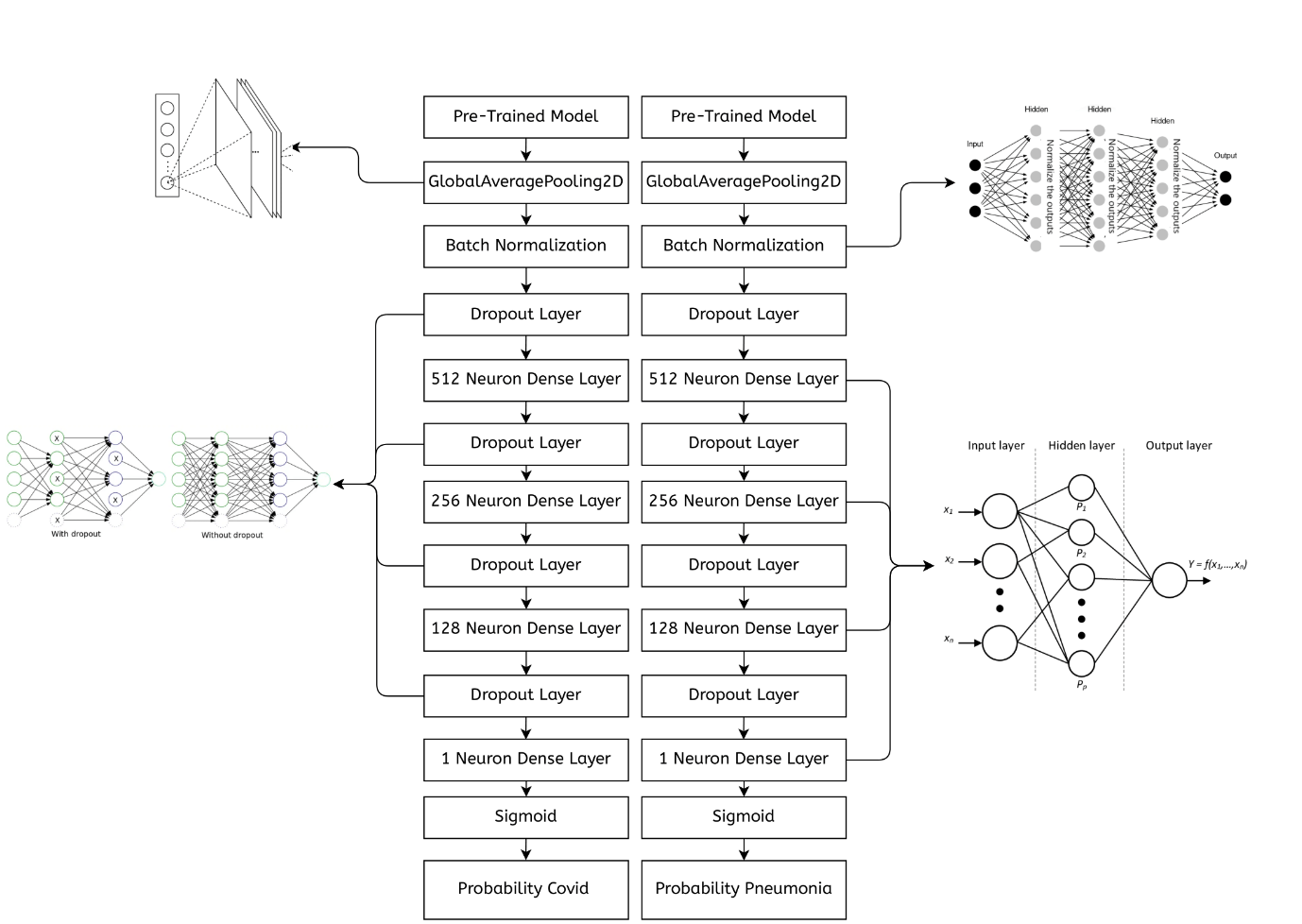


Here for our pretrained model Dense Net 169 was used for both branches as It was giving the best results which is show in our paper, CNNS were used as they have been shown to perform very well on images. Also because the other state of the art methods used CNNs we too decided to use CNNs. Our results show that CNNs were a good choice for our particular problem
